# Quorum Sensing N-acyl Homoserine Lactones-SdiA Suppresses *Escherichia coli*-*Pseudomonas aeruginosa* Conjugation through Inhibiting *traI* Expression

**DOI:** 10.3389/fcimb.2017.00007

**Published:** 2017-01-20

**Authors:** Yang Lu, Jianming Zeng, Binning Wu, Shunmei E, Lina Wang, Renxin Cai, Ni Zhang, Youqiang Li, Xianzhang Huang, Bin Huang, Cha Chen

**Affiliations:** ^1^Department of Laboratory Medicine, The Second Affiliated Hospital of Guangzhou University of Chinese MedicineGuangzhou, China; ^2^Postdoctoral Mobile Station, Guangzhou University of Chinese MedicineGuangzhou, China; ^3^The Second Clinical College, Guangzhou University of Chinese MedicineGuangzhou, China; ^4^Clinical Microbiology Laboratory, Guangdong Academy of Medical Science and Guangdong General HospitalGuangzhou, China; ^5^Department of Laboratory Medicine, The First Affiliated Hospital of Sun Yat-sen UniversityGuangzhou, China

**Keywords:** conjugation, N-acyl homoserine lactones, *P. aeruginosa*, SdiA, antibiotic resistance

## Abstract

Conjugation is a key mechanism for horizontal gene transfer and plays an important role in bacterial evolution, especially with respect to antibiotic resistance. However, little is known about the role of donor and recipient cells in regulation of conjugation. Here, using an *Escherichia coli* (*SM10*λπ)-*Pseudomonas aeruginosa* (*PAO1*) conjugation model, we demonstrated that deficiency of *lasI*/*rhlI*, genes associated with generation of the quorum sensing signals N-acyl homoserine lactones (AHLs) in *PAO1*, or deletion of the AHLs receptor SdiA in the donor *SM10*λπ both facilitated conjugation. When using another AHLs-non-producing *E. coli* strain *EC600* as recipient cells, deficiency of *sdiA* in donor *SM10*λπ hardly affect the conjugation. More importantly, in the presence of exogenous AHLs, the conjugation efficiency between *SM10*λπ and *EC600* was dramatically decreased, while deficiency of *sdiA* in *SM10*λπ attenuated AHLs-inhibited conjugation. These data suggest the conjugation suppression function of AHLs-SdiA chemical signaling. Further bioinformatics analysis, β-galactosidase reporter system and electrophoretic mobility shift assays characterized the binding site of SdiA on the promoter region of *traI* gene. Furthermore, deletion of *lasI*/*rhlI* or *sdiA* promoted *traI* mRNA expression in *SM10*λπ and *PAO1* co-culture system, which was abrogated by AHLs. Collectively, our results provide new insight into an important contribution of quorum sensing system AHLs-SdiA to the networks that regulate conjugation.

## Introduction

The acquisition of antibiotic resistance by pathogenic microorganisms is a threat to public health worldwide. Horizontal gene transfer, especially conjugative transfer of plasmids that carry resistance genes, is the primary cause of bacterial antibiotic resistance and—on the larger scale—bacterial evolution (Zatyka and Thomas, 1998; Arthur et al., 2011). The self-transmissible plasmids, such as the well-studied fertility F-plasmids and IncP plasmid RP4 (also known as RK2), generally present a mobilization (MOB) region which includes the origin of transfer (oriT) and the relaxase gene. The relaxase, identified as being TraI in RP4, initiates conjugation by cleaving the oriT in a site- and strand-specific manner (Carballeira et al., 2014). Other plasmids, termed mobilizable, are incapable of initiating conjugation, but can transfer by using the conjugative apparatus of another plasmid (Zatyka and Thomas, 1998). Mobilizable plasmids are more frequently found in natural environment; therefore, replication and mobilization can be considered as important mechanisms that influence plasmid promiscuity (Fernández-López et al., 2014).

Many Gram-negative bacteria utilize *N*-acyl homoserine lactones (AHLs) as signal molecules to enable individual bacteria to coordinate their behavior in populations; such quorum sensing (QS) enables bacteria to not only sense members of their own species but other species as well (Smith et al., 2011). The essential constituents of QS include a signal producer, or synthase, and a cognate transcriptional regulator that responds to the accumulated signal molecules (Bassler and Losick, 2006). The opportunistic animal and plant pathogen *Pseudomonas aeruginosa* possesses one of the best-studied models of QS, and two different AHL systems, *las* and *rhl*, have been identified (Wagner et al., 2003). In the *las* QS system, the *lasI* gene product directs formation of the diffusible extracellular signal *N*-(3-oxododecanoyl)-L-HSL (3-oxo-C12-HSL), which interacts with LasR to activate a number of virulence genes including the LasA and LasB elastases, exotoxinA, and alkaline protease (Toder et al., 1991; Gambello et al., 1993; Jones et al., 1993; Passador et al., 1993). In the *rhl* system, the *rhlI* gene product catalyzes the synthesis of *N*-butanoyl-L-HSL (C4-HSL). This diffusible signaling molecule, together with RhlR, activates directly some virulence genes like those encoding rhamnolipids and pyocyanin, and represses those genes responsible for assembly and function of the type III secretion system (Bleves et al., 2005; Jimenez et al., 2012). Besides the fact that the *las* and *rhl* systems are hierarchically connected, both *rhlR* and *rhlI* are positively regulated by the *las* system (Wagner et al., 2003). The roles of QS in diverse biological processes, such as virulence, biofilm formation and metabolism in *P. aeruginosa* have attracted research attention (Pearson et al., 1994; Hassett et al., 1999; Whiteley et al., 1999; García-Contreras, 2016). However, as the cell-to-cell communication system, the influence of QS on inter-species conjugation remains largely unknown.

Some organisms, such as *Escherichia, Klebsiella, Salmonella* and *Shigella* lack AHL synthase and therefore do not produce AHLs; however, they possess a LuxR homolog known as SdiA that can bind AHLs produced by other microorganisms and affect gene expression(Smith and Ahmer, 2003; Yao et al., 2006; Sabag-Daigle et al., 2015). Case et al. described the phenomenon of non-AHLs-producing microorganisms binding and utilizing AHLs produced by other organisms as eavesdropping (Case et al., 2008). Although SdiA can bind to DNA and regulate transcription in the absence of AHLs, the structural studies of SdiA suggest a double mode of action for AHLs on SdiA activity, by increasing both protein stability and DNA-binding affinity (Nguyen et al., 2015; Ishihama et al., 2016). Besides, a neighbor-joining tree analysis revealed that SdiA of *E. coli* did not cluster with the LuxR homologs found in other enterobacterial species, but was closely related to the RhlR of *P. aeruginosa* (Gray and Garey, 2001).

Herein, we clarified the effect of QS on conjugation and investigated the underlying mechanisms by employing a mobilizable plasmid and *E. coli-P. aeruginosa* conjugation model. We found that QS signal molecules produced by *P. aeruginosa* inhibited interspecies conjugation by activating *E. coli* SdiA, resulting in decreased mRNA expression of *traI* in *E. coli*. Blockade of AHL-SdiA signaling using strains deficient in *lasI, rhlI* or *sdiA* significantly enhanced conjugative transfer. These findings provide new insight into the regulatory networks of conjugation, and offer novel potential targets for antibiotic resistance.

## Materials and methods

### Bacterial strains, plasmids, and growth conditions

The bacterial strains and plasmids used in this study are listed in Table 1. Bacteria were grown in Lysogeny Broth (LB) medium or on LB plates containing 1.5% agar unless otherwise indicated. If required, antibiotics were added at the following final concentrations: ampicillin (Amp, 100 μg/mL), gentamycin (Gm, 30 μg/mL), chloramphenicol (Cm, 20 μg/mL), kanamycin (Kan, 50 μg/mL) and rifampicin (Rif, 50 μg/mL).

**Table 1:** **Bacterial strains and plasmids**.

**Strains/plasmids**	**Genotype or characteristics**	**Source**
**STRAINS**		
*E. coli SM10λπ*	thi thr leu tonA lacY supE recA::RP4-2-Tc::Mu Km λpir	Simon et al., 1983
*E. coli SM10λπ*Δ*sdiA*	Mutants of *E. coli SM10λπ* deficient in *sdiA* gene	This work
*E. coli EC600*	LacZ-, Nal^Rr^, Rif^R^	Our lab
*E. coli BW25113*	Δ(araD-araB)567, ΔlacZ4787(::rrnB-3), lambda-, rph-1, Δ(rhaD-rhaB)568, hsdR514	Our lab
*P. aeruginosa PAO1*	Wild-type	Stover et al., 2000
*PAO1*Δ*lasI*	Mutants of *PAO1* deficient in *lasI* gene	Our lab Zeng et al., 2016
*PAO1*Δ*rhlI*	Mutants of *PAO1* deficient in *rhlI* gene	Our lab Zeng et al., 2016
**PLASMIDS**		
pKD3	oriR6K, FRT::cat::FRT template plasmid Cm^R^, Amp^R^	Datsenko and Wanner, 2000
pKD46	oriR101 repA101ts P-araB-gam-bet-exo Amp^R^	Datsenko and Wanner, 2000
pCP20	pSC101 temperature-sensitive repliconts, Flp(λ Rp), cI857, Cm^R^, Amp^R^	Datsenko and Wanner, 2000
pQF50	Promoterless lacZ reporter plasmid, Amp^R^	Farinha and Kropinski, 1990
pQF50-*traI*	pQF50 derivative, containing *traI* promoter region, Amp^R^	This work
pUCP24T	370 bp oriT fragment from pCVD442 cloned into pUCP24, ori1600, Gm^R^	Philippe et al., 2004

*Rif^R^, Km^R^, Cm^R^, Gm^R^, and Amp^R^ stand for rifampicin, kanamycin, chloramphenicol, gentamycin and ampicillin resistance, respectively*.

### Growth curves

The indicated bacterial strains were cultured in LB overnight (8~10 h) at 37°C, then diluted to 0.5 MCF (McFarland standard) and 3 mL cultures were grown at 37°C with shaking at 200 rpm. The samples were collected at the indicated time points and the OD_600_ values were determined.

### Plasmid construction

The plasmid pUCP24T was constructed by inserting the *oriT* fragment into pUCP24 (West et al., 1994), which contains a gene cassette (*aacC1*) conferring gentamycin resistance in recipient cells. As a result, pUCP24T is not able to transfer on its own, but can transfer by using the conjugative apparatus of *E. coli SM10*λπ. Details of construction of the plasmids used to delete *sdiA* gene or express SdiA are described in the Supporting Materials and Methods.

### Construction of *PAO1 lasI* or *rhlI* and *E. coli SM10λπ sdiA* deficient mutants

The phage λ Red recombination system was employed for *sdiA* deletion in *E. coli SM10*λπ, while the *sacB*-based suicide vector system was adapted for knockout of *lasI* or *rhlI* in *PAO1* (Zeng et al., 2016); further details are provided in the Supporting Materials and Methods.

### Conjugation experiments

For the conjugation assays, the same amount (0.5 × 10^7^ CFU/mL, counted using the Sysmex UF-1000i™ Automated Urine Particle Analyzer; Tokyo, Japan) of mid-logarithmic phase donor (*E. coli SM10*λπ harboring plasmid pUCP24T) and recipient cells (*PAO1* or *EC600*) were mixed in 200 μL LB with or without the indicated HSLs in 96-well plates. After 6 h mating at 37°C, the cultures were vigorously mixed and 30 μL aliquots of each conjugation mixture were spread on LB agar containing 30 μg/mL Gm plus 100 μg/mL Amp for *SM10*λπ-*PAO1* or 30 μg/mL Gm plus 50 μg/mL Rif for *SM10*λπ-*EC600* transconjugants. The numbers of transconjugant colonies were counted after overnight incubation at 37°C.

### Quantification of HSLs by HPLc-MS/MS

Supernatants of *PAO1, PAO1*Δ*lasI*, and *PAO1*Δ*rhlI* cultures were collected for HPLC-MS/MS detection of HSLs; full details are provided in the Supporting Materials and Methods.

### β-galactosidase assays

β-Galactosidase activities were performed on cells in the mid-log phase of growth according to the modified Miller's method (Giacomini et al., 1992). All tests were performed in triplicate.

### Electrophoretic mobility shift assays (EMSA)

His-SdiA fusion protein was expressed in *E. coli BL21* (DE3) and purified via Ni-chelating affinity chromatography. Gel shift assays were carried out using the Lightshift Chemiluminescent EMSA kit according to the manufacturer's instructions (Thermo Scientific, Waltham, MA, USA), details are provided in the Supporting Materials and Methods.

### Real-time PCR

Total RNA was extracted using total RNA isolation reagent (Promega, Madison, WI, USA). Reverse transcription (1 μg of total RNA) was performed with the PrimeScript RT reagent Kit (Takara, Dalian, Liaoning, China). The cDNA was subjected to qPCR on a ViiA™ 7 Dx system (Applied Biosystems, Foster, CA, USA) using SYBR Green qPCR Master Mixes (ThermoFisher Scientific). The expression levels of the target genes were normalized to the expression of the internal control gene (*rpoD*), using the 2^−ΔΔCt^ method. The sequences of the primers are listed in Table S1.

### Statistical analysis

Data are expressed as the mean ± standard error of the mean (SEM) of at least three independent experiments. The differences between groups were analyzed using the Student's *t*-test when two groups were compared or one-way ANOVA when more than two groups were compared. All analyses were performed using GraphPad Prism, version 5 (GraphPad Software, Inc., San Diego, CA, USA). All statistical tests were two-sided; *P* < 0.05 was considered statistically significant.

## Results

### Deficiency of *lasI* or *rhlI* in *P. aeruginosa* promotes *SM10λπ-PAO1* conjugation

**Figure 1:**
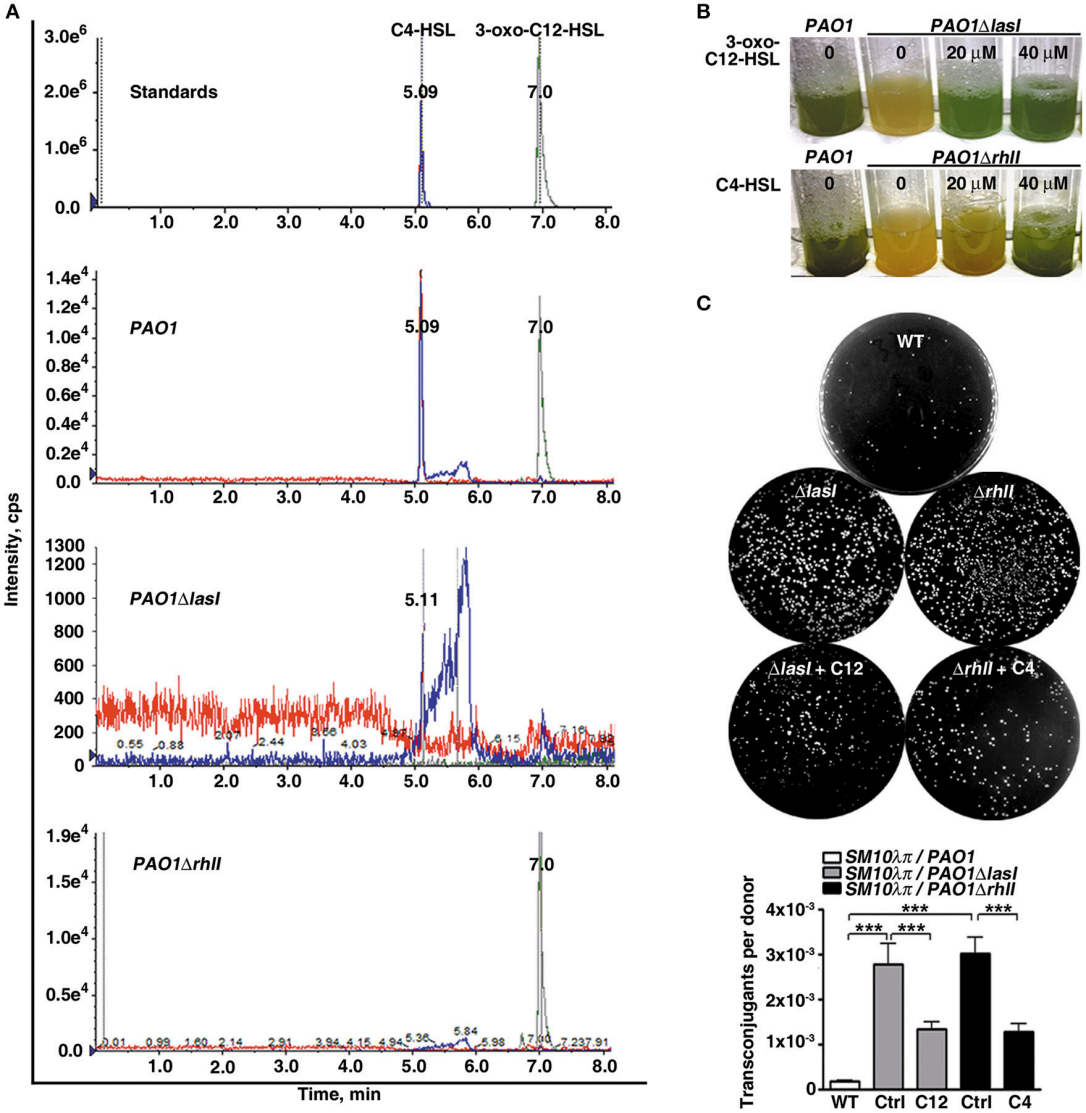
**The quorum sensing system of ***P. aeruginosa*** inhibits conjugation between ***P. aeruginosa*** and ***E. coli***. (A)** Deficiency of the AHLs synthase genes *rhlI* or *lasI* in *P. aeruginosa* (*PAO1*) resulted in the absence of C4-HSL or both 3-oxo-C12-HSL and C4-HSL, respectively. The 3-oxo-C12-HSL and C4-HSL in the cell-free supernatants were extracted with ethyl acetate and redissolved in methanol, followed by HPLC-MS/MS analysis. **(B)** Deficiency of *lasI* or *rhlI* in *P. aeruginosa* abolished production of the downstream toxin of *rhlI system* pyocyanin. *PAO1, PAO1*Δ*lasI* and *PAO1*Δ*rhlI* were cultured in the presence or absence of 3-oxo-C12-HSL or C4-HSL as indicated for 30 h. **(C)** Deficiency of *lasI* or *rhlI* in *P. aeruginosa* significantly promoted *SM10*λπ*-PAO1* conjugation; this effect could be abrogated by supplementation with exogenous 3-oxo-C12-HSL or C4-HSL. *SM10*λπ and *PAO1* (10^7^ CFU/mL each) were mated at 37°C for 6 h in the presence or absence of 40 μM of C4-HSL or 3-oxo-C12-HSL. Ctrl, control; C12, 3-oxo-C12-HSL; C4, C4-HSL. Values are mean ± SEM of at least three independent experiments; ^***^P < 0.001.

To elucidate the biological significance of the QS system in *P. aeruginosa* conjugation, we first constructed *lasI* or *rhlI* single gene-deficient mutants, named *PAO1*Δ*lasI* and *PAO1*Δ*rhlI*, respectively. In *P. aeruginosa, lasI* catalyzes the formation of 3-oxo-C12-HSL, which positively regulates the expression of RhlI. RhlI directs the synthesis of C4-HSL, which subsequently regulates pyocyanin production (O'Loughlin et al., 2013). In this study, despite the existence of *rhlI* in the genome of *PAO1*Δ*lasI*, both 3-oxo-C12-HSL and C4-HSL were barely detectable in the conditioned medium of this mutant strain using HPLC-MS/MS analysis. For *PAO1*Δ*rhlI*, the deficiency of *rhlI* in the genome led to an absence of C4-HSL in the conditioned medium of this mutant strain, whereas *lasI* and its product 3-oxo-C12-HSL were present at similar levels as the WT strain (Figure 1A and Figure S1). Furthermore, as a result of mutation of the QS system, both *PAO1*Δ*lasI* and *PAO1*Δ*rhlI* lost the ability to express pyocyanin, which could be rescued by exogenous addition of 3-oxo-C12-HSL or C4-HSL (Figure 1B). Taken together, these results confirmed the successful creation of *PAO1* strains deficient in *lasI* or *rhlI*.

We subsequently examined the growth and conjugation ability of *PAO1*Δ*lasI* and *PAO1*Δ*rhlI*. Compared to the WT strain, deficiency of *lasI* or *rhlI* hardly affected the growth of *PAO1* (Figure S2), but significantly promoted *SM10*λπ*-PAO1* conjugation (Figure 1C). Furthermore, exogenous 3-oxo-C12-HSL or C4-HSL attenuated the interspecies conjugation ability of *PAO1*Δ*lasI* and *PAO1*Δ*rhlI* (Figure 1C). What's more, we counted the amount of donor *SM10*λπ after co-culture with *PAO1, PAO1*Δ*lasI* or *PAO1*Δ*rhlI*, and found that there is no difference among the three groups (Figure S3), indicted that the observed effect of quorum sensing on conjugation efficiency was not due to the growth suppressive effect on *SM10*λπ. These data suggested that the QS system may negatively regulate *SM10*λπ*-PAO1* conjugation.

### The quorum sensing system of *P. aeruginosa* inhibits conjugation by activating SdiA of *E. coli*

**Figure 2:**
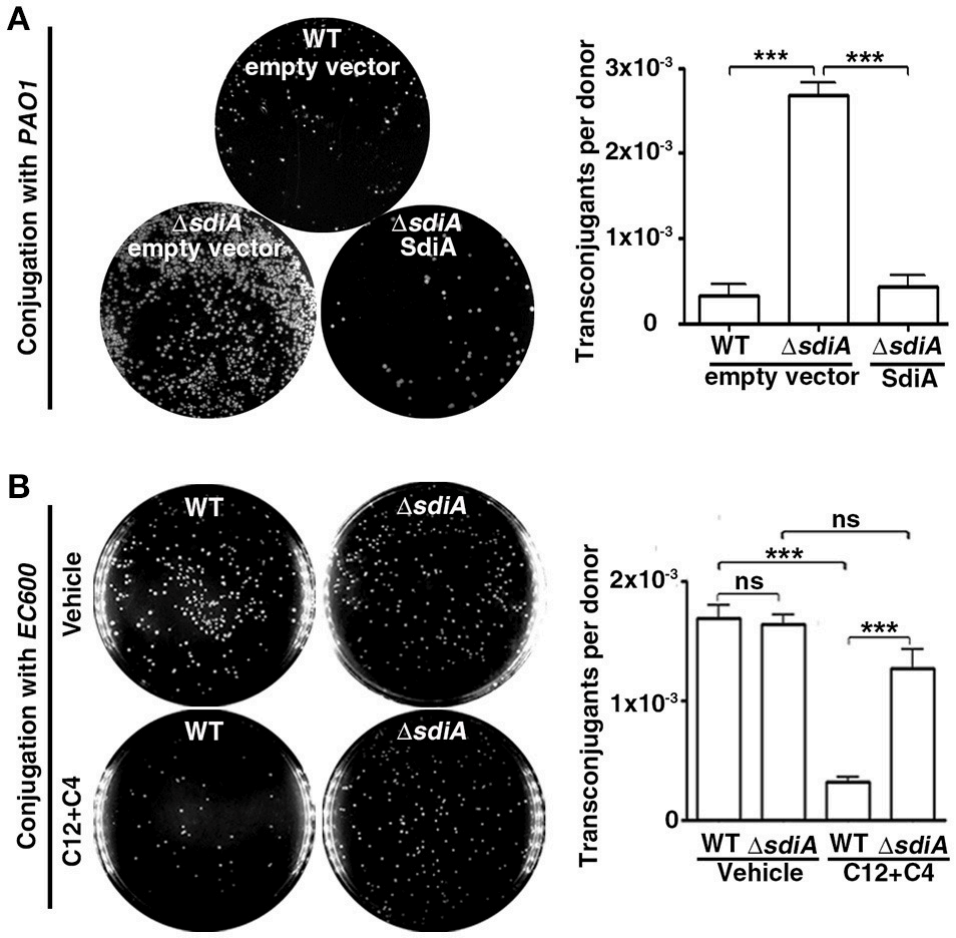
**AHL inhibits ***SM10***λπ-***PAO1*** conjugation via a mechanism dependent on SdiA of ***E. coli***. (A)** Deficiency of *sdiA* in *E. coli* significantly promoted *SM10*λπ*-PAO1* conjugation. *E. coli SM10*λπ and *P. aeruginosa PAO1* (10^7^ CFU/mL each) were mated at 37°C for 6 h. **(B)** Deficiency of *sdiA* in *SM10*λπ did not significantly affect *SM10*λπ*-EC600* conjugation, but could rescue 3-oxo-C12-HSL and C4-HSL (C4/12)-inhibited conjugation. *SM10*λπ was cultured in the presence of DMSO or 40 μM C4-HSL and 3-oxo-C12-HSL for 6 h, followed by conjugation with *EC600*. Values are mean ± SEM of at least three independent experiments; ns, not significant, ^***^P < 0.001.

It is well recognized that AHLsq regulate gene transcription via binding to their receptor proteins (LuxR-like proteins). In this conjugation model, in contrast to the recipient cells *PAO1*, the donor *E. coli SM10*λπ cells lack AHL synthase and therefore do not produce AHLs; however, these cells produce a LuxR homolog known as SdiA that can bind AHLs produced by other bacterial species to regulate gene transcription. Given that the conjugative apparatus exist in donor cells, we speculated that *P. aeruginosa*-released AHLs may act on SdiA of *E. coli*. To assess whether SdiA of *E. coli* is involved in the ability of *P. aeruginosa*'s AHLs to inhibit *E. coli*-*P. aeruginosa* conjugation, we constructed the *sdiA* deficient mutant *SM10*λπΔ*sdiA*. As expected, deficiency of *sdiA* in *SM10*λπ significantly enhanced *E. coli-P. aeruginosa* conjugation, whereas overexpression of SdiA reversed the phenotype (Figure 2A). However, when using a AHLs-non-producing *E. coli* strain *EC600* as the recipient cell, *SM10*λπΔ*sdiA* did not increase conjugation ability compared to the WT strain (Figure 2B). More importantly, the conjugation efficiency of *SM10*λπ and *EC600* significantly decreased in the presence of exogenous 3-oxo-C12-HSL and C4-HSL, while *sdiA* deletion in *SM10*λπ abrogated the effects of AHLs on conjugation (Figure 2B), suggesting the inhibitory effect of SdiA on *E. coli-P. aeruginosa* or *SM10*λπ*-EC600* conjugation is dependent on the presence of AHLs. In addition, growth curves demonstrated that deficiency of *sdiA* in *E. coli* had no influence on cell proliferation (Figures S4, S5), confirming that the regulatory function of SdiA in conjugation in this model was not due to an altered growth rate.

Collectively, these data imply that AHLs produced by *PAO1* may repress *SM10*λπ-*PAO1* conjugation through binding to SdiA of *E. coli*.

### The interaction between *P. aeruginosa* HSL and *E. coli* SdiA inhibits the expression of *traI* in *E. coli*

**Figure 3:**
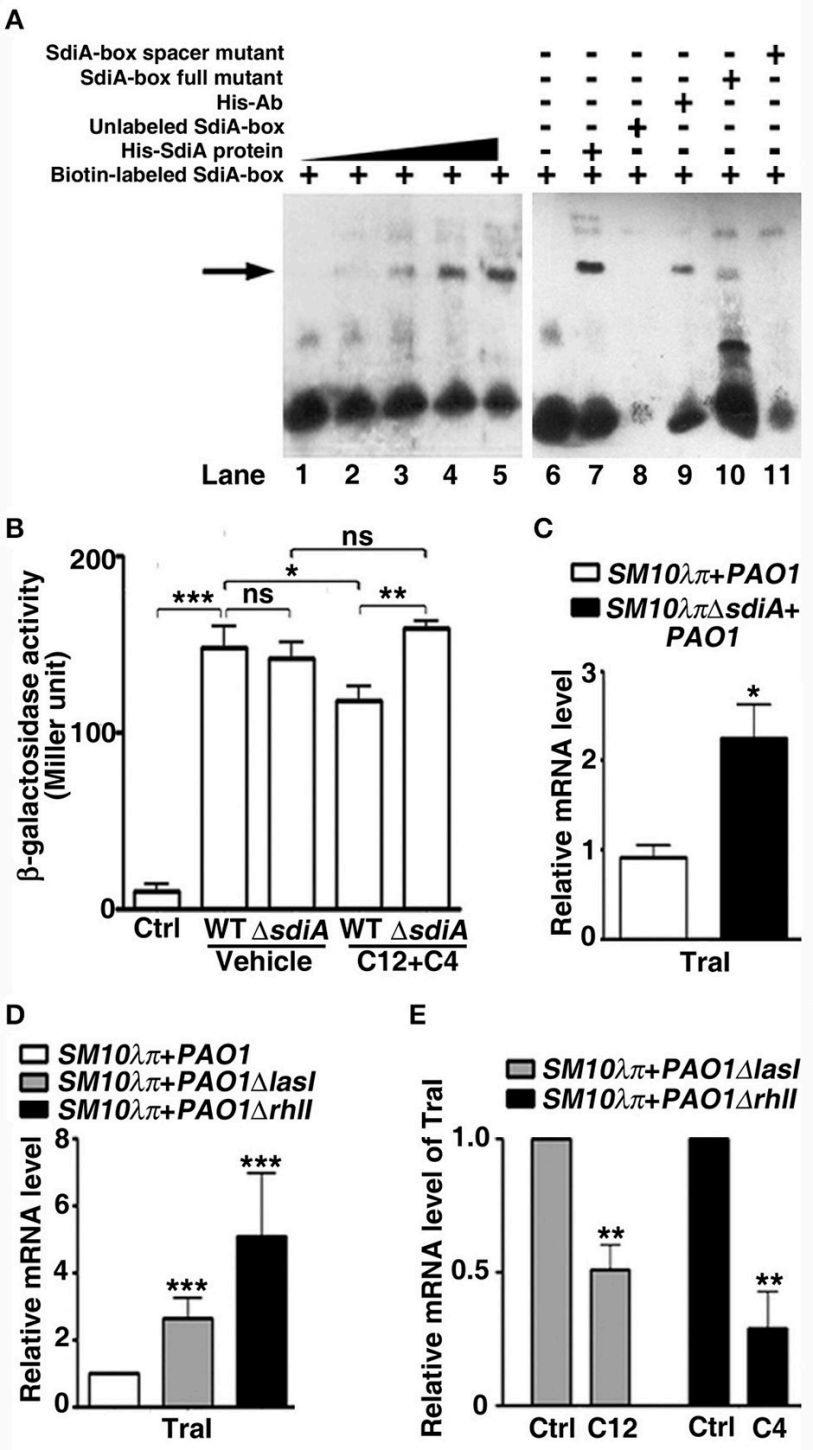
**The AHL-SdiA interaction represses the expression of ***TraI*** in ***E. coli***. (A)** EMSA verified the interaction of SdiA protein with the putative SdiA-box of the *traI* promoter. Biotin-labeled SdiA-box (50 fmol) was added to each reaction with 0, 2, 4, 8, or 16 pmol *E. coli* His-SdiA fusion protein (lane 1 to lane 5, respectively). The specificity analysis (lane 6 to lane 11) was performed in the presence (−) or absence (+) of His-SdiA fusion protein (8 pmol) and biotin-labeled SdiA-box (50 fmol, lanes 7), 200-fold unlabeled SdiA-box (lanes 8), anti-His antibody (lanes 9), or 200-fold SdiA-box full or spacer mutant competitors (lane 10, 11). **(B)** Activity of β-galactosidase reporters containing the predicted SdiA-box in genomic regions upstream of *traI* under various conditions. *E.coli BW25113* or *E.coli BW25113*Δ*sdiA* were cultured with *SM10*λπ in the presence of DMSO (Ctrl) or 40 μM C4-HSL and 3-oxo-C12-HSL for 6 h, followed by β-galactosidase activity analysis. **(C)** Deficiency of *sdiA* promoted *traI* expression in *E. coli*. **(D)** Deficiency of *lasI/rhlI* promoted *traI* expression in *E. coli*. For **(C,D)**, 10^7^ CFU/mL (each) of the indicated *E. coli* donor and recipient *P. aeruginosa* cells were mated at 37°C for 6 h, followed by real-time PCR analysis. The *rpoD* gene of *E. coli* was used as an internal control. **(E)** Exogenous 3-oxo-C12-HSL or C4-HSL inhibited *traI* expression in *E. coli*. *PAO1*Δ*lasI* or *PAO1*Δ*rhlI* were cultured with *SM10*λπ in the presence of DMSO (Ctrl) or 40 μM C4-HSL and 3-oxo-C12-HSL for 6 h, followed by real-time PCR analysis. The *rpoD* gene of *E. coli* was used as an internal control. Ctrl, control; C12, 3-oxo-C12-HSL; C4, C4-HSL. Values are mean ± SEM of at least three independent experiments; ^*^P < 0.05; ^**^P < 0.01; ^***^P < 0.001.

Mechanisms behind transcription regulation function of SdiA is being disclosed, it seems that genes with specific DNA sequences (SdiA-box) 5′-AAAAG(N8)GAAAA-3′ in the promoter region may be the potential targets of SdiA (Yamamoto et al., 2001). In view of the presence of SdiA-box in the promoter of many SdiA-regulated genes in our bioinformatics analysis (Table S2), we computationally mapped the DNA sequence in the RP4 plasmid to search for conjugation-related genes potentially regulated by SdiA. An SdiA-box sequence (5′-AAGAGcgattgagGAAAA-3′) was identified −317 bp upstream of the *traI* start codon (Figure S6). Subsequently, EMSA assays confirmed the interaction between SdiA and the predicted SdiA-box of the *traI* promoter *in vitro* (Figures 3A,B). We therefore further evaluated the role of SdiA in the regulation of *traI* transcription. DNA fragments of *traI* promoter carrying the predicted SdiA-box was cloned upstream of the β -galactosidase gene in the pQF50-promoter reporter. When transformed into *BW25113* (another *E. coli* strain without endogenous β-galactosidase compared to *SM10*λπ), the β-galactosidase activity of pQF50-*traI* was greatly elevated, compared to that of the control, while addition of 3-oxo-C12-HSL and C4-HSL impaired this activity, which was severely attenuated when the *sdiA* was deleted (Figure 3B). Intriguing, when AHLs was absent, deletion of *sdiA* hardly affected β-galactosidase activity of pQF50-*traI* (Figure 3B), this is consistent with the phenotype shown in Figure 2. Compared with the WT strain, *SM10*λπΔ*sdiA* showed higher mRNA expression of *traI* when cultured with *PAO1* (Figure 3C). On the other hand, in the *SM10*λπ*-PAO1* co-culture system, deficiency of *lasI* or *rhlI* in *PAO1* also led to enhanced expression of *traI* (Figure 3D), while supplementation with exogenous 3-oxo-C12-HSL and C4-HSL significantly repressed *traI* expression (Figure 3E). These results suggest that repressing *traI* expression in the donor cells may be a critical mechanism behind the inhibitory effect of the AHLs on conjugation.

In summary, we disclosed the cooperative effect of AHLs produced by recipient *P. aeruginosa* cells and SdiA of donor *E. coli* cells in the conjugation regulation. These findings indicate that QS may inhibit conjugation and prevent the excessive dissemination of plasmid.

## Discussion

Most recent publications in this field have focused on the regulatory function of QS in virulence and biofilm formation. Here, using *E. coli* (*SM10*λπ) as donor cells and AHLs-producing *P. aeruginosa* (*PAO1*) or non-AHLs producing *E. coli* (*EC600*) as recipient cells, we identified a conjugation-inhibitory effect for QS based on the following evidence. First, for *SM10*λπ and *PAO1* co-culture system in which AHLs is normally self-sustained, deficiency of the AHLs-producing genes *lasI* or *rhlI* in *PAO1* or the solo AHLs receptor SdiA in *SM10*λπ promoted *SM10*λπ-*PAO1* conjugation, while supplementation with exogenous 3-oxo-C12-HSL or C4-HSL abrogated the enhanced conjugation ability of *PAO1*Δ*lasI* and *PAO1*Δ*rhlI*. On the other hand, for both non-AHLs producing *SM10*λπ and *EC600* mixed cultures, stimulation with exogenous 3-oxo-C12-HSL and C4-HSL inhibited conjugation, while deletion of *sdiA* in *SM10*λπ attenuated this effect. Conventionally, conjugation is considered to be mainly regulated by the self-transmissible plasmids. While our results indicate that QS system of donor and recipient cells may play a role in conjugation regulation.

Conjugation enables the dissemination of virulence genes and antibiotic resistance genes, which leads to the adaption of bacteria to new circumstances (Norman et al., 2009). Therefore, the ability to inhibit conjugation may be a potentially efficacious strategy for avoiding the spread of resistance traits. Here, we demonstrate that AHL-SdiA is capable of suppressing conjugation. Most SdiA-expression bacteria, such as *Escherichia, Salmonella* and *Shigella* are enterobacteria, while many biological evidences suggest a lack of HSLs in the normal mammalian intestine (Swearingen et al., 2013), despite the presence of AHLs in bovine rumen (Hughes et al., 2010). Thus, although *P. aeruginosa* could be detected in stool sample in our clinical microbiology laboratory, future studies are needed to illuminate the role of AHL-SdiA signaling in pathogenic bacteria communities within the gastrointestinal tract.

To date, many SdiA regulon members have been described (Kanamaru et al., 2000; Wei et al., 2001; Dyszel et al., 2010; Sabag-Daigle et al., 2015). Here we report the identification of SdiA-regulated and AHL-responsive gene *traI* in the plasmid RP4. TraI is reported to function as a relaxase enzyme that creates a nick at the *oriT* of conjugative plasmids, which is required to initiate conjugation (Furuya and Komano, 2000). We discovered a DNA motif recognized by SdiA in the promoter region of the *traI* gene in the plasmid RP4, and the interaction between SdiA and the predicted SdiA-box was validated *in vitro* using an EMSA. However, some SdiA-regulated genes do not have this particular SdiA-box (Dyszel et al., 2010; Swearingen et al., 2013; Abed et al., 2014; Nguyen et al., 2015), there may be other conjugation-related genes repressed by AHL-SdiA. Moreover, the EMSA was performed without addition of AHLs, so it seems that high concentration of SdiA could bind to *traI* promoter in the absence of AHLs *in vitro* (Figure 3A), however, the reporter system (Figure 3B) and conjugation experiment (Figure 2B) showed that in the absence of AHLs, deletion of *sdiA* hardly affected the promoter activity of *traI*, as well as conjugation frequency *in vivo*. Thus, we proposed AHLs may increase both SdiA protein stability and *traI* promoter-binding affinity to repress *traI* expression.

Despite the advantages of conjugation for bacteria, the introduction of novel genes into the pre-existing, well-tuned genetic background is a source of genetic conflict, and possession of the conjugation-associated machinery also places a burden on the host arising from the energy expended to create and maintain the conjugative apparatus and its associated features (Zatyka and Thomas, 1998; Baltrus, 2013; San Millan et al., 2015). This raises the question of how host bacteria minimize the metabolic cost while obtaining the benefits provided by conjugation. In this study, we found that under normal conditions, when mobilizable plasmid containing a resistance gene was not required by *PAO1* (Table S3), conjugation between *SM10*λπ and *PAO1* was inhibited via the LasI/RhlI-HSL-SdiA pathway. These findings reveal that QS system may play a role in protecting host cells against external conjugative plasmids.

Utilizing ecological data from 2801 samples, Freilich et al. explored the ubiquitous competitive and cooperative interactions between the bacteria within natural communities (Freilich et al., 2011). Nonetheless, revealing more detail of the strategies bacteria adopt for survival in mixed cultures remains a major challenge. The *E. coli-P. aeruginosa* conjugation model has been widely used in studies of bacterial conjugation, and the most prevalent donor strain is *E. coli SM*λπ in which the RP4 plasmid is chromosomally-integrated. Thus, conjugation-associated genes, such as *traI* initially only exist in and are expressed by the *E. coli* (*SM10*λπ) cells, similarly *rhlI* and *lasI* are only expressed by *PAO1*. This makes it easy to detect the expression of these genes in *E. coli* (*SM10*λπ) and *PAO1*, specifically in mixed-cultures. Using this co-culture system, we found that LasI/RhlI and SdiA jointly repressed *traI* expression in *E. coli* and inhibited *SM10*λπ-*PAO1* conjugation, indicating that the QS system may provide a mechanism of cooperative regulation between bacteria.

In conclusion, the findings of this study highlight the regulatory role for the QS system in conjugation, and expand our understanding of the bacterial communication and defense systems of *P. aeruginosa*.

## Author contributions

YL, JZ, XH, BH, and CC designed research; YL, JZ, BW, RC, and NZ performed research; SE and YQL contributed new reagents/analytic tools; YL, JZ, and BW analyzed data; YL, JZ, and CC wrote the paper.

## Funding

This work was supported by the National Natural Science Foundation of China (Grant No. 81572058, 81672081), the Natural Science Foundation of Guangdong Province (Grant No. 2014A030313143) and the Science and Technology Planning Project of Guangdong Province (Grant No. 2016A020215236).

### Conflict of interest statement

The authors declare that the research was conducted in the absence of any commercial or financial relationships that could be construed as a potential conflict of interest.

## Abbreviations

QS: quorum sensing
AHLs: N-acyl homoserine-lactones
*P. aeruginosa*: *Pseudomonas aeruginosa*
*E. coli*: *Escherichia coli*
qPCR: quantitative real-time PCR.
