# Supplementary figures and images for: Quorum Sensing N-acyl Homoserine Lactones-SdiA Suppresses Escherichia coli-Pseudomonas aeruginosa Conjugation through Inhibiting traI Expression
Source: Front Cell Infect Microbiol. 2017 Jan 20;7:7. doi: 10.3389/fcimb.2017.00007 (PMC5247672; doi:10.3389/fcimb.2017.00007)

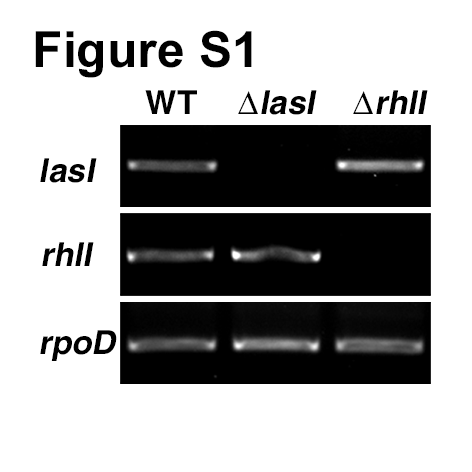

Supplement: Supplementary file 1 [file Image1.TIF]

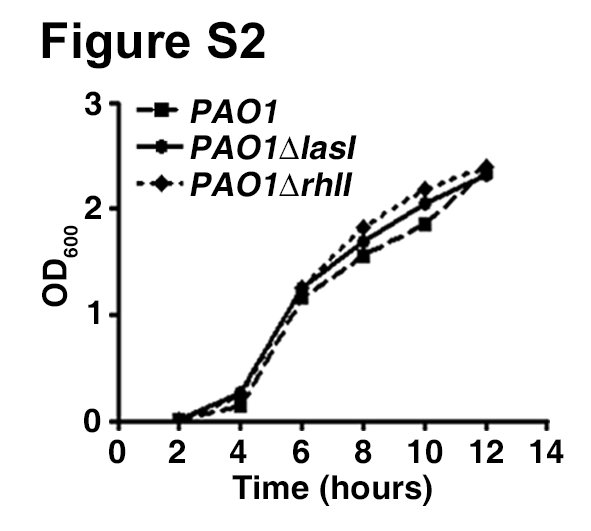

Supplement: Supplementary file 2 [file Image2.TIF]

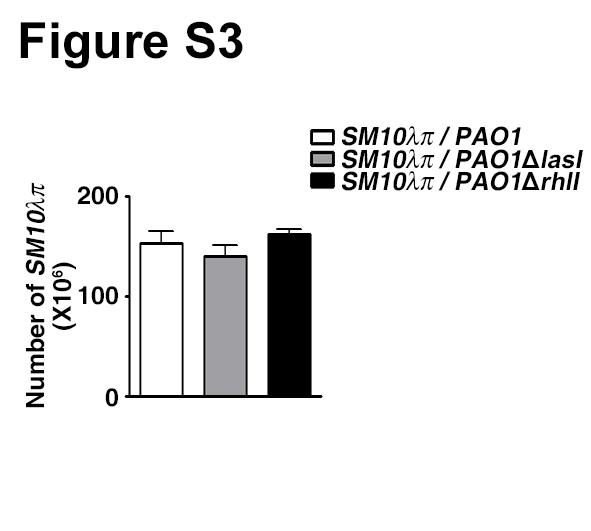

Supplement: Supplementary file 3 [file Image3.TIF]

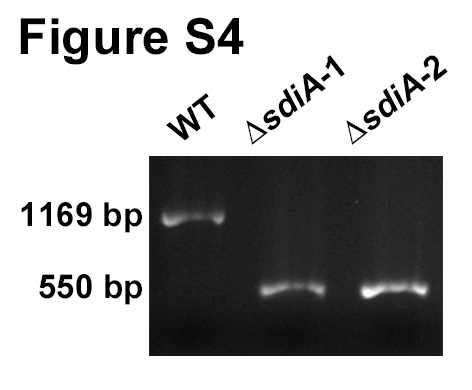

Supplement: Supplementary file 4 [file Image4.TIF]

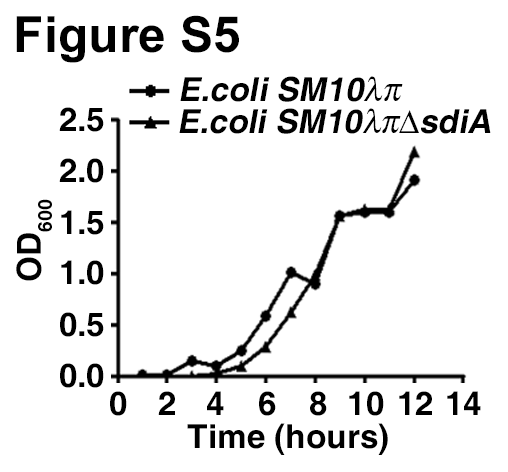

Supplement: Supplementary file 5 [file Image5.TIF]

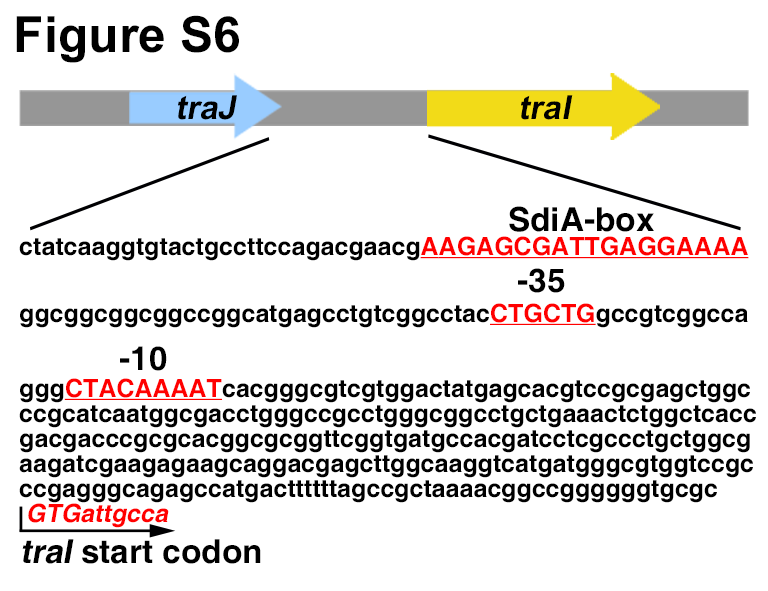

Supplement: Supplementary file 6 [file Image6.TIF]
