# Supplementary material for: Quorum Sensing N-acyl Homoserine Lactones-SdiA Suppresses Escherichia coli-Pseudomonas aeruginosa Conjugation through Inhibiting traI Expression
Source: Front Cell Infect Microbiol. 2017 Jan 20;7:7. doi: 10.3389/fcimb.2017.00007 (PMC5247672; doi:10.3389/fcimb.2017.00007)
Supplement: Supplementary file 7 [file Table1.DOC]

| **Table S1.** Sequences of RNA and DNA oligonucleotides | | |
| --- | --- | --- |
| **Name** | **Sense primer (5'-3')** | **Antisense primer (5'-3')** |
| **Primers for qPCR** |  |  |
| *traI* | ATCACGAAGAAGGGAACCATCATC | TTGAACTCTGCTGTGCCGTTGAC |
| *rpoD (**E. Coli)* | TATCTGCTGGAACAGTACGATCGTG | TGTTGTCATCATCGGCGCTG |
| *lasI* | CGTGCTCAAGTGTTCAAGGA | AAAACCTGGGCTTCAGGAGT |
| *rhlI* | CTACCGGCATCAGGTCTTCA | GTTTCGCTGCACAGGTAGG |
| *rpoD (P. aeruginosa)* | CTGAAGATCGCCAAAGAGCC | GTGTGGTCGGTGTTCATGTC |
| **Primers for *traI* promoter cloning** |  |  |
| *traI* | GCTCTAGACATCGTCTCTCGCCTGTCCCCT | GCTCTAGACACGCGCACCCCCCG |
| **Probes for EMSA** |  |  |
| SdiA-box | biotin-ACGAACGAAGAGcgattgagGAAAAGGCGGCGGCG | CGCCGCCGCCTTTTCctcaatcgCTCTTCGTTCGT |
