# Supplementary material for: Quorum Sensing N-acyl Homoserine Lactones-SdiA Suppresses Escherichia coli-Pseudomonas aeruginosa Conjugation through Inhibiting traI Expression
Source: Front Cell Infect Microbiol. 2017 Jan 20;7:7. doi: 10.3389/fcimb.2017.00007 (PMC5247672; doi:10.3389/fcimb.2017.00007)
Supplement: Supplementary file 8 [file Table2.DOC]

**Table S2.** the predicted SdiA-box on the promoter region of literature-reported genes.

| No. | **Gene** | **Predicted SdiA-box** | genome | Distance-CDS (bp) | Strand/location | Source |
| --- | --- | --- | --- | --- | --- | --- |
| 1 | ***ftsQAZ*** | **AAAAGtagtagcaGAAAA** | *E. coli* | 458 | +1/102696 | [4] |
| 2 | ***pheA*** | **AAGATCGTCTGGCGAAAT** | *E. coli* | 1088 | +1/2734678 | [4] |
| 3 | ***pheP*** | **AAAAGCGATAGCTGAAAA** | *E. coli* | 723 | +1/600458 | [4] |
| 4 | ***uvrY*** | **AAAATTTGCGGTGAAAAA** | *E. coli* | 1072 | -1/1993798 | [5] |
| 5 | ***ydiV*** | **AAAAGgacccctgAAAAA** | *E. coli* | 824 | -1/1790154 | [6] |
| 6 | ***gadW*** | **GAAAGacgtaaatGAAAT** | *E. coli* | 216 | -1/3662128 | [7] |
| 7 | ***gadE*** | **AAAAGtgctgtggGAAAG** | *E. coli* | 551 | +1/3655819 | [7] |
| 8 | ***yhiD*** | **AGAAAAACCCGCAGAAAA** | *E. coli* | 111 | -1/3654035 | [7] |
| 9 | ***hdeA*** | **ATAAAATTAAGAAGAAAA** | *E. coli* | 225 | -1/3654988 | [7] |
| 10 | ***hdeB*** | **AAAAGCAGCTGATAACAA** | *E. coli* | 356 | -1/3654671 | [7] |
| 11 | ***fliE*** | **AAAAGgggcgcgcGTACA** | *E. coli* | 654 | +1/2011692 | [7] |
| 12 | ***acrA*** | **AAAATGTCCAGGAAAAAT** | *E. coli* | 808 | -1/485651 | [8] |
| 13 | ***csgD*** | **aaaattgtgcaataaaaa** | *E. coli* | 276 | -1/1102695 | [9] |
| 14 | ***srgA*** | **AAACGcataaccgGAATA** | *pSLT* | 900 | -1/7438 | [10] |
| 15 | ***srgA*** | **GAAAGtgaatgccGGAAA** | *pSLT* | 1396 | -1/7934 | [10] |
| 16 | ***srgC*** | **AGAATaattccctGAAAA** | *pSLT* | 164 | +1/4889 | [10] |
| 17 | ***ybbK*** | **AAAGTACGTTGTTGAAAa** | *S.typhimurium* | 281 | +1/562620 | [10] |
| 18 | ***adrA*** | **aaaggcgatgcgcgtaaa** | *S.typhimurium* | 259 | +1/437851 | [10] |
| 19 | ***ybbJ*** | **GAAAGCCGAGGGGGAAAA** | *S.typhimurium* | 303 | -1/561728 | [10] |
