# Supplementary material for: Quorum Sensing N-acyl Homoserine Lactones-SdiA Suppresses Escherichia coli-Pseudomonas aeruginosa Conjugation through Inhibiting traI Expression
Source: Front Cell Infect Microbiol. 2017 Jan 20;7:7. doi: 10.3389/fcimb.2017.00007 (PMC5247672; doi:10.3389/fcimb.2017.00007)
Supplement: Supplementary file 9 [file Table3.DOC]

| **Table S3.** Comparison of plasmid stability using plate dilution method | | | | | | | |
| --- | --- | --- | --- | --- | --- | --- | --- |
| **Cultivation**  **Time (hour)** | ***PAO1* (pUCP24T)**  **with selective pressure（presence of Gm）** | | |  | ***PAO1* (pUCP24T)**  **without selective pressure（absence of Gm）** | | |
|  | **CFU with**  **selective plate** | **CFU with nonselective plate** | **Plasmid stability (%)** |  | **CFU with**  **selective plate** | **CFU with nonselective plate** | **Plasmid stability (%)** |
| 12 | 287 | 295 | 97.29 |  | 308 | 312 | 98.72 |
| 24 | 371 | 385 | 96.36 |  | 307 | 341 | 90.03 |
| 36 | 320 | 336 | 95.24 |  | 175 | 185 | 94.59 |
| 48 | 355 | 371 | 95.69 |  | 210 | 236 | 88.98 |
| 60 | 215 | 218 | 98.62 |  | 463 | 516 | 89.73 |
| 72 | 325 | 330 | 98.48 |  | 299 | 370 | 80.81 |
| 84 | 410 | 431 | 95.13 |  | 247 | 389 | 63.50 |
| 96 | 253 | 259 | 97.68 |  | 104 | 290 | 35.86 |
| 108 | 230 | 234 | 98.29 |  | 74 | 340 | 21.76 |
