# Supplementary material for: Quorum Sensing N-acyl Homoserine Lactones-SdiA Suppresses Escherichia coli-Pseudomonas aeruginosa Conjugation through Inhibiting traI Expression
Source: Front Cell Infect Microbiol. 2017 Jan 20;7:7. doi: 10.3389/fcimb.2017.00007 (PMC5247672; doi:10.3389/fcimb.2017.00007)
Supplement: Supplementary file 10 [file DataSheet1.DOC]

***Supplementary Material***

**Quorum sensing N-acyl homoserine lactones-SdiA suppresses *Escherichia coli*-*Pseudomonas aeruginosa* conjugation through inhibiting traI expression**

**Yang Lu**^1,2†^**,** **Jianming** **Zeng**^1†^**,** **Binning Wu**^1^**,** **Shunmei E**^1^**,** **Lina Wang**^1^**, Renxin Cai**^1^**,** **Ni Zhang**^3^**,** **Youqiang Li**^1^**,** **Xianzhang Huang**^1,2^**,** **Bin Huang**^4*^ **and Cha Chen**^1*^

*** *Correspondence:*** *Cha Chen, chencha906@163.com;* *Bin Huang,* *huangb3@mail.sysu.edu.cn.*

**Supplementary Materials and Methods**

**Plasmids construction**

For construction of SdiA-expressing plasmid, SdiA coding region was amplified by PCR form *E. coli* SM10λpir chromosomal DNA. Amplified fragments were purified by gel extraction kit (DongSheng, Xian, Shanxi, China), then digested with *Hind III* and *BamH I* (Takara, Dalian, Liaoning, China), and cloned into the corresponding sites of pET32a (+).

**Construction of** ***E. coli* *SM10λπ* *sdiA* deficient mutants**

Construction of *E. coli* *SM10λπ* *sdiA* mutant followed the same steps described by Datsenko *et al* [1] except 100 mM L-arabinose was used to induce *P_araB_* promoter of pKD46. Briefly, the insert fragments were amplified with primer D-*sdiA*-F (ATGCAGGATAAGGATTTTTTCAGCTGGCGTCGCACGATGTAGGCTGGAGCTGCTTCG) and D-*sdiA*-R (TCAAATTAAGCCAGTAGCGGCCGCGTAACAGGCAACCATGGGAATTAGCCATGGTCC). Afterwards, 25 μL cells were transfected with 300-500ng PCR products using electroporation according to the manufacturer's instructions. Recombinants were selected on LB agar (Cm, 15μg/mL) and then transformed with pCP20 to eliminate FRT-flanked *cat* gene. Both mutants were verified by PCR (primers C-*sdiA*-F: TTTCAAGCCTGATAAAACTGC and C-*sdiA*-R: AGAATAATCAAGAACTGGCAAA) and DNA sequencing.

**Quantification of HSL by HPLC-MS/MS**

After clarification by centrifugation, 1 mL of the cell-free supernatant was extracted three times with an equal volume of ethyl acetate, which was supplemented with 0.2 mol/L acetic acid to acidify it [2]. Thereafter, the combined organic phase was dried in an N_2_ stream, redissolved in 1 mL methanol and stored at -20°C. Twenty microliters of each sample was added to 980 μL of methanol to dilute by 50-fold and filtered through 0.22 μm nylon disk filters immediately prior to HPLC-MS/MS analysis [3]. Ten microliters of the sample was used for HPLC-MS/MS analysis. Six-point standard curves were generated for C4-HSL and 3-oxo-C12-HSL, and the standard mixture was reanalyzed after every sixth sample. The standards N-butyryl-L-HSL (C4-HSL) and N-(3-oxododecanoyl)-L-HSL (3-oxo-C12-HSL) were purchased from Sigma. Methanol, acetonitrile (HPLC grade), and formic acid (MS grade) were purchased from Fisher Scientific (Loughborough, UK). Acetic acid and ethyl acetate (HPLC grade) were obtained from Guangzhou Chemical Reagent Factory (Guangzhou, China) and ultra-pure water was obtained from Milli-Q water Elga Maxima water purification system (Merck, KgaA, Germany).

**Electrophoretic mobility shift assays (EMSA)**

His-SdiA fusion protein was expressed in *E. coli* BL21 (DE3) and purified via Ni-chelating affinity chromatography. Gel shift assays were carried out using the Lightshift Chemiluminescent EMSA kit according to the manufacturer’s instructions (Thermo Scientific, Waltham, MA, USA), details are provided in the Supporting Materials and Methods.The recombined His-SdiA-expressing plasmid was transformed into *E. coli* BL21 (DE3) strains (Novagen, Darmstadt, Germany). Thereafter, the transformants were cultured in 200 mL LB containing 1 mM IPTG for 8 h at 30°C with shaking at 200 rpm until the cell density reached an OD600 of 0.6. The cells were harvested by centrifugation, lysed by sonication and centrifuged at 12,000 rpm for 30 min at 4°C. The supernatant was purified via Ni-chelating affinity chromatography and the purity of SdiA was assessed by SDS-PAGE and quantified using a modified Bradford protein assay kit (Sangon Biotech, Shanghai, China). Gel shift assays were carried out using the Lightshift Chemiluminescent EMSA kit according to the manufacturer’s instructions (Thermo Scientific, Waltham, MA, USA). The labeled DNA fragments (50 fmol) were incubated with various amounts of purified His-SdiA at room temperature for 20 min and and His-antibody or 200-fold molar excess of unlabeled WT or mutant SdiA-box fagements as competitor. SdiA-DNA complexes were separated by 6% PAGE in 0.5×TBE buffer. The biotin-labeled DNA (listed in S1) was then transferred to a nylon membrane and detected using a chemiluminescence assay.

**Supplementary References:**

1. Datsenko KA, Wanner BL. One-step inactivation of chromosomal genes in Escherichia coli K-12 using PCR products. Proc Natl Acad Sci U S A. 2000;97: 6640–6645.

2. Charlton TS, de Nys R, Netting A, Kumar N, Hentzer M, Givskov M, et al. A novel and sensitive method for the quantification of N-3-oxoacyl homoserine lactones using gas chromatography-mass spectrometry: application to a model bacterial biofilm. Environ Microbiol. 2000;2: 530–541.

3. Ortori CA, Dubern J-F, Chhabra SR, Cámara M, Hardie K, Williams P, et al. Simultaneous quantitative profiling of N-acyl-L-homoserine lactone and 2-alkyl-4(1H)-quinolone families of quorum-sensing signaling molecules using LC-MS/MS. Anal Bioanal Chem. 2011;399: 839–850.

4. Yamamoto K, Yata K, Fujita N, Ishihama A. Novel mode of transcription regulation by SdiA, an Escherichia coli homologue of the quorum-sensing regulator. Mol Microbiol. 2001;41: 1187–1198.

5. Wei Y, Lee JM, Smulski DR, LaRossa RA. Global impact of sdiA amplification revealed by comprehensive gene expression profiling of Escherichia coli. J Bacteriol. 2001;183: 2265–2272.

6. Zhou X, Meng X, Sun B. An EAL domain protein and cyclic AMP contribute to the interaction between the two quorum sensing systems in Escherichia coli. Cell Res. 2008;18: 937–948.

7. Dyszel JL, Soares JA, Swearingen MC, Lindsay A, Smith JN, Ahmer BMM. E. coli K-12 and EHEC genes regulated by SdiA. PLoS One. 2010;5: e8946.

8. Rahmati S, Yang S, Davidson AL, Zechiedrich EL. Control of the AcrAB multidrug efflux pump by quorum-sensing regulator SdiA. Mol Microbiol. 2002;43: 677–685.

9. Lee J, Maeda T, Hong SH, Wood TK. Reconfiguring the quorum-sensing regulator SdiA of Escherichia coli to control biofilm formation via indole and N-acylhomoserine lactones. Appl Environ Microbiol. 2009;75: 1703–1716.

10. Ahmer BM, van Reeuwijk J, Timmers CD, Valentine PJ, Heffron F. Salmonella typhimurium encodes an SdiA homolog, a putative quorum sensor of the LuxR family, that regulates genes on the virulence plasmid. J Bacteriol. 1998;180: 1185–1193.

**Supplementary Figure Legends**

S1 Figure. Identification of the specific deficiency of *lasI* and *rhlI* in *PAO1*. The genomic DNA preparations of *PAO1*, *PAO1*Δ*lasI* and *PAO1*Δ*rhlI* were subjected to PCR assays.

S2 Figure. Deficiency of *rhlI* or *lasI* hardly affected the growth of *PAO1*. The curve of bacteria growth was shown.

S3 Figure. Deficiency of *rhlI* or *lasI* in *PAO1* hardly affected the growth of *SM10λπ* in co-culture system. *E. coli SM10λπ* and *PAO1,* *PAO1ΔlasI* or *PAO1ΔrhlI* (107 CFU/mL each) were mated at 37ºC for 6 h.

S4 Figure. Gene fragment of *sdiA* was deleted in *SM10λπ.* The genomic DNA preparations of *SM10λπ* and *SM10λπ*Δ*sdiA* were subjected to PCR assays.

S5 Figure. Deficiency of *sdiA* hardly affected the proliferation of *SM10λπ*. The growth curve of *SM10λπ* and *SM10λπ*Δ*sdiA* was shown*.*

S6 Figure. Diagram of the traI promoter in the RP4 plasmid. The putative SdiA-box, -10 and -35 boxes are indicated in red.
